# Chikungunya Vaccines in Travel Medicine: From Regulatory Approval to Real-World Challenges

**DOI:** 10.3390/tropicalmed11080221

**Published:** 2026-08-07

**Authors:** Yann Hervigo, Cornelia Staehelin, Olivia Veit, François Chappuis, Gilles Eperon, Stefano Musumeci

**Affiliations:** 1 Division of Internal Medicine, Fribourg Hospital, 1752 Villars-sur-Glâne, Switzerland; 2 Department of Infectious Diseases, Inselspital, Bern University Hospital, University of Bern, 3010 Bern, Switzerland; 3 Swiss Tropical and Public Health Institute, 4123 Allschwil, Switzerland; 4 University of Basel, 4001 Basel, Switzerland; 5 Division of Tropical and Humanitarian Medicine, Geneva University Hospitals, 1205 Geneva, Switzerlandgilles.eperon@hug.ch (G.E.); 6 Faculty of Medicine, University of Geneva, 1205 Geneva, Switzerland; 7 Division of Infectious Diseases, Geneva University Hospitals, 1205 Geneva, Switzerland

**Keywords:** chikungunya, chikungunya vaccines, travel medicine

## Abstract

**Background:** Chikungunya virus (CHIKV) infection is an emerging disease of growing concern to international travelers due to the expanding geographic distribution of competent vectors, climate change, and frequent outbreaks in endemic and previously unaffected regions. Until recently, prevention relied exclusively on vector control and bite prevention; however, the recent approval of chikungunya vaccines has introduced a new preventive strategy. **Methods:** We conducted a narrative review of currently licensed chikungunya vaccines and candidates in clinical development, focusing on immunogenicity, safety, regulatory status, and implications for travel medicine practice. **Results:** Two vaccines have recently received regulatory approval: the live-attenuated vaccine IXCHIQ^®^ and the virus-like particle vaccine Vimkunya^®^. Both demonstrated high immunogenicity based on neutralizing antibody thresholds accepted as surrogate markers of protection. Post-marketing safety signals associated with IXCHIQ^®^, particularly in older adults, have led to divergent regulatory decisions between authorities. The indication of vaccination in national recommendations varies somewhat depending on age, duration of travel, and individual risk factors. Several additional vaccine platforms remain under investigation, although some candidates have been discontinued. **Conclusions:** The recent approval of chikungunya vaccines marks a significant milestone in the field of travel medicine. However, uncertainties regarding long-term protection, safety in specific populations, and optimal targeting of travelers remain. Individualized risk–benefit assessment is essential, and further data are needed to refine vaccination strategies for travelers and populations at risk.

## 1. Introduction

Chikungunya virus (CHIKV) is an arbovirus transmitted by *Aedes albopictus* and *Aedes aegypti* [1]. First isolated in Tanzania in 1952, it initially caused sporadic outbreaks. However, the emergence of the East-Central-South African (ECSA) strain in Kenya in 2004 triggered a major epidemic that spread across the Indian Ocean islands, where the Indian Ocean lineage (IOL) emerged. Subsequently, the epidemic spread to Asia and India [2]. CHIKV is now endemic or associated with epidemics in nearly 100 countries worldwide [3].

Four genotypes have been identified, West African, Asian, ECSA, and IOL, the latter two currently predominating [4]. Despite this genetic diversity, only one serotype is recognized [5]. The global spread of chikungunya has been driven by urbanization, globalization and international travel, climate change, and viral adaptation, enhancing transmission by *Aedes albopictus*, a vector now widely distributed across tropical, subtropical, and increasingly temperate regions, including Europe [6,7].

From January to early December 2025, more than 500,000 cases were reported to the World Health Organization including 208,335 confirmed cases and 186 deaths worldwide, underscoring its ongoing public health impact [7]. In Europe, although most cases are travel-related, autochthonous transmission has been documented since 2007 in Italy and 2010 in mainland France, with both countries experiencing their largest outbreaks to date in 2025 (384 and 788 locally acquired cases, respectively) [8].

Clinical manifestations of CHIKV infection include an acute febrile illness characterized by high fever, headache, myalgia, rash, and severe inflammatory polyarthralgia, with symptom prevalence varying by age group. The extremes of age experience higher rates of hospitalization and mortality. Older adults (>65 years) are at increased risk of severe or chronic arthralgia, while perinatal infection carries a significant risk of vertical transmission of approximately 50% and high rates of neurological complications among infected neonates [9,10]. Although mortality is low, chronic joint involvement is common, persisting for more than three months in approximately 43% and up to 12 months in 21% of patients [11]. The global disease burden remains substantial, with an estimated annual loss of 106 000 Disability-Adjusted Life Years (DALYs) worldwide between 2010 and 2019 [12].

Despite numerous studies evaluating candidate antiviral molecules, with some showing reductions in viral titers, as for other arboviral infections, no specific antiviral treatment has proven effective against chikungunya [13]. Clinical management therefore remains supportive, relying on antipyretics, analgesics, fluid therapy, and rest, while non-steroidal anti-inflammatory drugs should only be used after exclusion of dengue infection due to hemorrhagic risk [14,15]. The absence of specific antiviral therapy further underscores the importance of prevention.

Until recently, personal prevention relied exclusively on mosquito bite prevention such as repellents and mosquito nets. In 2024, two chikungunya vaccines were approved by both the U.S. Food and Drug Administration (FDA) and the European Medicines Agency (EMA). A modelling study estimated that vaccination campaigns could avert 4436 infections and 17 DALYs per 100,000 doses administered [3].

The limited availability of data from endemic regions and the unpredictable nature of outbreaks have hindered the feasibility of large-scale efficacy trials. Consequently, regulatory approval was granted based on surrogate markers of protection, with protective neutralizing antibody thresholds defined using passive serum transfer experiments in the non-human primate models and supported by seroepidemiological studies [16]. Despite the recent increase in reported cases, clinical efficacy data remain unavailable. These thresholds should be interpreted cautiously, as their predictive value across different populations (including endemic and non-endemic settings), viral lineages and long-term protection remain uncertain. Moreover, the two approved vaccines rely on distinct neutralization assay-systems to define these surrogate endpoints, limiting direct comparison of their immunogenicity data (Table 1). This narrative review aims to present currently approved vaccines, discuss other vaccine platforms in development, and explore their implications for travel medicine.

## 2. Approved Chikungunya Vaccines for Clinical Use

### 2.1. IXCHIQ^®^ (VLA1553) Vaccine

IXCHIQ^®^ (VLA1553) manufactured by Valneva^®^ was the first chikungunya vaccine to be licensed. It is a live-attenuated virus vaccine carrying the Δ5nsP3 deletion, where nsP3 is an essential protein for viral replication. The vaccine strain is derived from an ECSA/IOL genotype chikungunya virus isolated during the 2006 outbreak in La Réunion [17].

Following two phase III clinical trials, IXCHIQ^®^ received accelerated approval by the FDA in November 2023, and subsequently by the EMA in June 2024. The vaccine is delivered as a single intramuscular dose of 0.5 mL [18].

#### 2.1.1. Immunogenicity

A protective threshold was defined as a CHIKV-specific neutralising antibody titre of ≥150 as measured by the micro-plaque reduction neutralisation test (µPRNT50) at 28 days post-vaccination, based on passive transfer studies in non-human primates [19]. In a phase III trial, the per-protocol immunogenicity analysis showed that 98.9% (263/266) of VLA1553 recipients achieved seroprotective chikungunya virus neutralizing antibody titers 28 days after a single vaccination, with comparable responses across age groups [20]. Sustained immunogenicity was confirmed in a phase 3b study, with seroprotection rates at 2 years of 96.8% (18–64 years) and 98% (≥65 years) [21]. Seroprotection rates at 3 years were at 96.4% and still at 94.5% at 4 years after a single dose [22]. In adolescents (12–17 years old), seroprotection reached 98.8% at 28 days and 98.3% at 12 months [23]. Lot-to-lot consistency was also demonstrated [24].

#### 2.1.2. Pre-Marketing Safety

In a pooled safety study, in participants receiving the vaccine (*n* = 3520), 63.7% reported adverse reactions, the majority of which were mild. The most common local reaction was injection-site tenderness (11%), while systemic reactions included headache (31.9%), fatigue (29.5%), and myalgia (23.8%). Arthralgia was reported in 16.7% of cases. Two serious adverse events were linked to the vaccination, both of which resolved completely following hospitalization [25]. Safety profiles were similar across age groups, including adolescents [23,25]. Chikungunya-like reactions, defined as fever (≥38 °C) with associated symptoms, occurred in 11.7% of participants within 30 days after vaccination [26].

#### 2.1.3. Post-Marketing Safety and Regulatory Actions

In early 2025, a large-scale vaccination campaign using IXCHIQ^®^ was conducted during a major outbreak in La Réunion, with approximately 40,000 doses deployed, primarily targeting older adults and individuals at higher risk of severe disease [27]. Post-marketing surveillance identified serious adverse events, predominantly among older adults with comorbidities (91% of cases; median age 73 years). Individuals aged ≥65 years received approximately 33% of administered vaccine doses but accounted for 77% of reported adverse events, with most events occurring within 7 days post-vaccination. Reported events included chikungunya-like syndromes, as well as neurological, cardiac, and renal manifestations. By 31 August 2025, 35 serious adverse events had been reported, including three fatal outcomes. One fatal case involved encephalitis with detection of the vaccine strain in cerebrospinal fluid [28].

In response to these safety signals, both the FDA and EMA implemented temporary restrictions in May 2025 for older adults (>59 years for FDA; ≥65 years for EMA) [29,30]. Following reassessment, the EMA lifted restrictions in July 2025, recommending vaccination based on individual benefit–risk evaluation, particularly given the higher risk of severe disease in older populations [29]. In contrast, the FDA, through the Center for Biologics Evaluation and Research (CBER), first lifted the restriction in July 2025 and then concluded that the benefit–risk balance was unfavorable and suspended the IXCHIQ^®^ license on 22 August 2025 [30]. In January 2026, Valneva^®^ finally announced the voluntary withdrawal of its Biologics License Application (BLA) and Investigational New Drug (IND) applications in the United States for its IXCHIQ^®^ vaccine [31].

A case of aseptic meningitis in a previously healthy young individual following administration of IXCHIQ^®^ was reported to the EMA’s Pharmacovigilance Risk Assessment Committee (PRAC). Following review, PRAC included aseptic meningitis among the known adverse reactions associated with IXCHIQ^®^, alongside encephalitis and encephalopathy. The committee’s conclusions will be further reflected in the Periodic Safety Update Report (PSUR), expected to be finalized in June 2026 [32].

#### 2.1.4. Ongoing and Planned Studies

Several post-authorization studies are ongoing. A phase III trial is evaluating antibody persistence for up to 10 years [33]. Another is assessing safety and immunogenicity in children aged 1–11 years in multiple countries in Latin America and Southeast Asia [34]. A third study is investigating coadministration with dengue vaccines [35].

### 2.2. Vimkunya^®^ (VRC-CHKVLP059-00-VP/PXVX0317)

Vimkunya^®^ manufactured by Bavarian Nordic^®^ is a virus-like particle (VLP) vaccine composed of the chikungunya virus capsid and envelope proteins derived from the CHIKV strain 37,997 (West African lineage). The absence of non-structural proteins prevents viral replication while preserving antigenic structure. The formulation includes aluminum hydroxide as an adjuvant to enhance the immune response [36]. Vimkunya^®^ received accelerated approval from the FDA on 14 February 2025, for use in adolescents aged ≥12 years and adults, followed by EMA approval on 28 February 2025. The vaccine is delivered as a single intramuscular 0.8 mL dose. The need of a booster is still being evaluated on immunogenicity trials [37,38].

#### 2.2.1. Immunogenicity

Immunogenicity was assessed using neutralisation assays, with a protective serum neutralising antibody (SNA) titre defined as ≥1:100 (reciprocal dilution), derived from sero-epidemiological data and supported by non-human primate studies [39,40]. Two phase III trials evaluated immunogenicity across age groups. In participants aged 12–64 years (*n* = 2559), seroresponse reached 97.8% at 3 weeks and declined to 85.5% at 6 months [41]. In adults aged ≥65 years (*n* = 206), seroresponse was lower, reaching 87% at 3 weeks and 76% at 6 months. Despite this decline, most older participants maintained antibody titres above the seroprotection threshold at 6 months [42].

#### 2.2.2. Safety

Vimkunya^®^ demonstrated a favorable safety profile across all age groups evaluated in phase 3 trials. Most adverse events were mild to moderate, transient, and included injection-site pain, fatigue, headache, and myalgia. Similar tolerability was observed in adults aged ≥ 65 years, with no vaccine-related serious adverse events or deaths reported, thus supporting the vaccine’s use across a broad age range [38,41,42].

#### 2.2.3. Ongoing and Planned Studies

Two phase III trials are currently underway: one evaluating safety and immunogenicity in children aged 2–11 years, and another assessing long-term immunity for up to five years following a single dose in individuals aged ≥ 12 years [41,42,43]. Additional studies are planned in pregnant individuals and in children aged <12 years, in line with post-authorisation requirements from the FDA [37].

**Table 1:** Key characteristics of licensed chikungunya vaccines.

Characteristic	IXCHIQ^®^ (VLA1553)	Vimkunya^®^ (PXVX0317)
Platform	Live-attenuated CHIKV (Δ5nsP3 deletion)	Recombinant virus-like particle (VLP), adjuvanted
Dose	Single 0.5 mL intramuscular dose	Single 0.8 mL intramuscular dose
Age indication	≥12 years (EU)	≥12 years
Correlate of protection	µPRNT50 ≥ 150	Serum neutralizing antibody titre ≥ 1:100
Immunogenicity	98.9% seroprotection at Day 28; 94.5% at Year 4	97.8% seroresponse at Week 3 (12–64 y); 87% in ≥65 y; 85.5% and 76% at Month 6
Safety	Mostly mild-to-moderate reactogenicity; post-marketing serious adverse events predominantly in older adults with comorbidities	Mostly mild-to-moderate reactogenicity; no vaccine-related serious adverse events reported in Phase III studies
Contraindications	Pregnancy, immunocompromised individuals, severe hypersensitivity to vaccine components	Severe hypersensitivity to vaccine components
Regulatory status	EMA approved; FDA licence withdrawn	FDA- and EMA-approved

## 3. Other Vaccine Platforms Under Trials

Several alternative vaccine platforms are under investigation, although progress remains heterogeneous and some programs have been discontinued (Table 2).

### 3.1. BBV87

BBV87 is a β-propiolactone-inactivated whole-virion CHIKV vaccine adjuvanted with aluminum hydroxide. It has completed phase I evaluation [44,45]. While an initial phase II/III study was terminated, another trial is ongoing in India, and preclinical data support protective efficacy in non-human primates [46,47].

### 3.2. ChAdOx1 Chik

This chimpanzee adenovirus-vectored vaccine demonstrated 100% seroconversion in early phase I studies [48,49]. However, no further clinical development has been reported.

### 3.3. MV-CHIK

A measles virus-vectored vaccine candidate showed high immunogenicity in phase I/II trials, with up to 100% seroconversion after booster dosing and cross-neutralizing activity against multiple CHIKV strains [50,51,52,53,54]. Despite promising Phase I and II trials, the industrial partner terminated the program. Consequently, the Institut Pasteur reclaimed the asset and announced in June 2026 a European Union-funded Phase Ib/III trial enrolling participants aged 5 to 55 [55].

### 3.4. VAL-181388

This is an mRNA-based vaccine candidate that demonstrated acceptable safety and durable humoral responses up to one year in a phase I trial [56,57]. No further clinical development has been announced.

### 3.5. HydroVax-005 CHIKV

This is a hydrogen peroxide-inactivated vaccine candidate showing protective efficacy in preclinical models [58]. A phase I clinical trial is currently ongoing [59].

### 3.6. CHIKV TSI-GSD-218

This is an early live-attenuated vaccine candidate that demonstrated immunogenicity in phase I/II trials but was discontinued due to concerns regarding genetic stability [60,61].

## 4. Travel Medicine Recommendations

Chikungunya vaccination recommendations for travelers vary across countries, reflecting differences in regulatory decisions, vaccine availability, and national risk tolerance. However, these variations remain relatively limited and largely rely on three core determinants: the definition of outbreak situations, the duration of stay in at-risk areas, and the risk of occupational exposure, particularly among laboratory personnel. The vaccination is recommended for individuals in the situation listed below if they are aged 12 or older for Vimkunya. Regarding IXCHIQ, vaccination is recommended from 12 years old and the maximum age recommendation varies in Europe between 59 and 64 years old.

Firstly, travel to areas experiencing an outbreak represents the most consistent indication for vaccination across all national guidelines. Although this criterion may seem straightforward at first glance, discrepancies may arise from differing definitions and surveillance methods for outbreaks. These vary depending on the data sources used (e.g., national surveillance systems, CDC, or ECDC reports).

Secondly, the duration of stay in endemic or high-risk areas is a key factor in guiding whether vaccination is necessary. Although the threshold for the length of stay varies slightly between countries, ranging from a few weeks to several months, the underlying principle remains consistent: prolonged exposure increases the cumulative risk of infection and may justify vaccination, particularly for individuals at a higher risk of developing severe or chronic CHIKV disease.

Thirdly, occupational exposure, especially among laboratory workers handling CHIKV, is uniformly recognized as an indication for vaccination. Recommendations for this group are highly consistent across countries.

**Figure 1:**
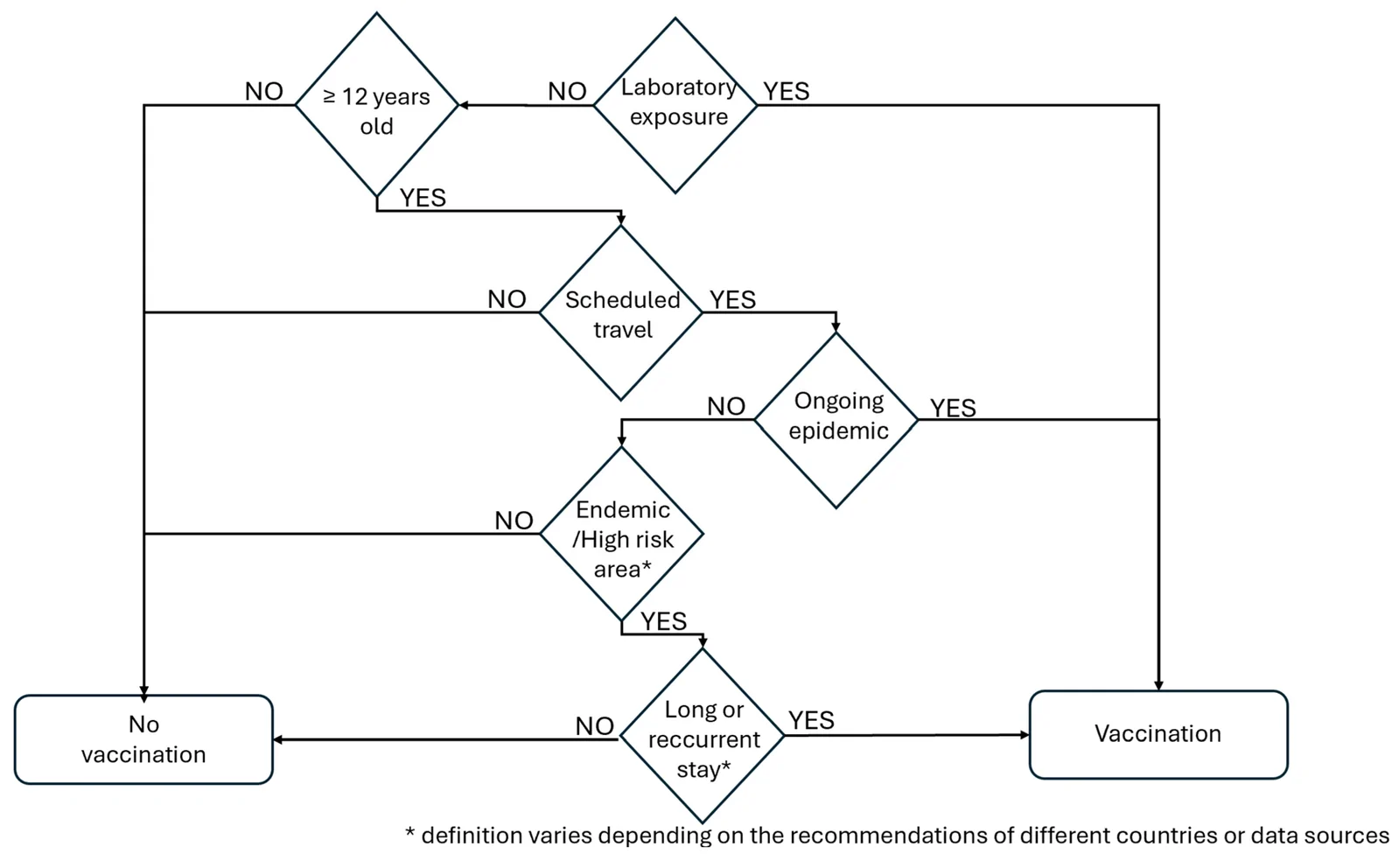
Clinical decision pathway for chikungunya vaccination in individuals at risk of CHIKV exposure.

Overall, the differences between national recommendations are modest and primarily reflect variations in the definition of outbreaks and the interpretation of epidemiological data, rather than fundamentally different strategies (Figure 1). Efforts are currently underway by international scientific societies to harmonize these definitions and, consequently, vaccination recommendations. In practice, travel medicine decisions continue to rely on an individualized risk–benefit assessment, taking into account traveler characteristics, destination-specific epidemiology, and evolving safety data, particularly regarding the live-attenuated vaccine IXCHIQ^®^ [62,63,64,65,66].

## 5. Conclusions

Recently approved chikungunya vaccines represent an important advance for travel medicine and may expand preventive options for travelers and populations at risk. However, uncertainties remain regarding long-term protection, vaccine safety in specific populations, and the optimal identification of individuals most likely to benefit from vaccination. Given the evolving evidence base and differences in national recommendations, vaccination decisions should rely on an individualized risk–benefit assessment. Further post-marketing and effectiveness data will be essential to refine vaccination strategies and define the role of chikungunya vaccines in travel medicine practice. Public health and regulatory authorities will need to address key implementation challenges, including ensuring equitable and affordable access to the vaccines in endemic countries, integrating vaccination into travel medicine schedules, and addressing vaccine safety and use.

## Figures and Tables

**Table 2:** Clinical development status of licensed and investigational Chikungunya vaccines (legend: green indicates a completed clinical phase; blue indicates an ongoing clinical phase; red indicates discontinued clinical development; ND indicates that no publicly available data were identified regarding the current development status).

	Vaccine Platform	Phase I	Phase II	Phase III	Phase IV
IXCHIQ^®^	Live-attenuated				
Vimkunya^®^	Virus-like particle				ND
BBV87	β-propiolactone-inactivated whole-virion				
ChAdOx1 Chik	Adenovirus-vectored		ND		
MV-CHIK	Measles virus-vectored				
VAL-181388	mRNA-based		ND		
HydroVax-005 CHIKV	Hydrogen peroxide-inactivated				
CHIKV TSI-GSD-218	Live-attenuated				

## Data Availability

No new data were created or analyzed in this study. Data sharing is not applicable to this article.
